# C-MEMS-derived glassy carbon electrochemical biosensors for rapid detection of SARS-CoV-2 spike protein

**DOI:** 10.1038/s41378-023-00601-4

**Published:** 2023-11-06

**Authors:** Naresh Mandal, Raja Mitra, Bidhan Pramanick

**Affiliations:** 1 School of Electrical Sciences, Indian Institute of Technology Goa, 403401 Ponda, Goa India; 2 School of Chemical and Materials Sciences, Indian Institute of Technology Goa, 403401 Ponda, Goa India; 3 Centre of Excellence in Particulates Colloids and Interfaces, Indian Institute of Technology Goa, 403401 Ponda, Goa India; 4 School of Interdisciplinary Life Sciences, Indian Institute of Technology Goa, 403401 Ponda, Goa India

**Keywords:** Biosensors, Electrical and electronic engineering, Environmental, health and safety issues

## Abstract

According to a World Health Organization (WHO) report, the world has experienced more than 766 million cases of positive SARS-CoV-2 infection and more than 6.9 million deaths due to COVID through May 2023. The WHO declared a pandemic due to the rapid spread of the severe acute respiratory syndrome coronavirus 2 (SARS-CoV-2) virus, and the fight against this pandemic is not over yet. Important reasons for virus spread include the lack of detection kits, appropriate detection techniques, delay in detection, asymptomatic cases and failure in mass screening. In the last 3 years, several researchers and medical companies have introduced successful test kits to detect the infection of symptomatic patients in real time, which was necessary to monitor the spread. However, it is also important to have information on asymptomatic cases, which can be obtained by antibody testing for the SARS-CoV-2 virus. In this work, we developed a simple, advantageous immobilization procedure for rapidly detecting the SARS-CoV-2 spike protein. Carbon-MEMS-derived glassy carbon (GC) is used as the sensor electrode, and the detection is based on covalently linking the SARS-CoV-2 antibody to the GC surface. Glutaraldehyde was used as a cross-linker between the antibody and glassy carbon electrode (GCE). The binding was investigated using Fourier transform infrared spectroscopy (FTIR) characterization and cyclic voltammetric (CV) analysis. Electrochemical impedance spectroscopy (EIS) was utilized to measure the change in total impedance before and after incubation of the SARS-CoV-2 antibody with various concentrations of SARS-CoV-2 spike protein. The developed sensor can sense 1 fg/ml to 1 µg/ml SARS-CoV-2 spike protein. This detection is label-free, and the chances of false positives are minimal. The calculated LOD was ~31 copies of viral RNA/mL. The coefficient of variation (*C*V) number is calculated from EIS data at 100 Hz, which is found to be 0.398%. The developed sensor may be used for mass screening because it is cost-effective.

**Figure:**
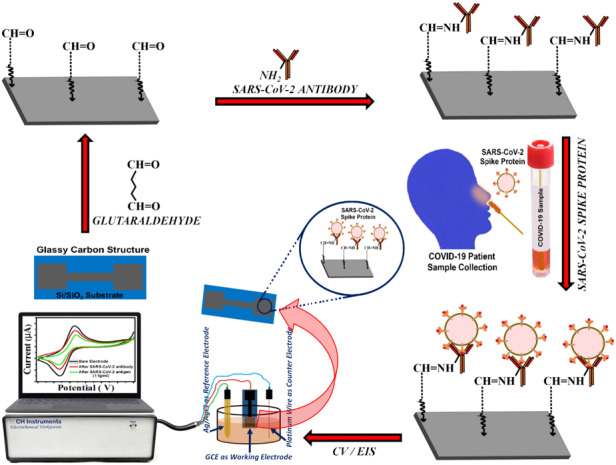
A schematic representation of the SARS-CoV-2 spike protein sensing using surface functionalized glassy carbon electrode

A schematic representation of the SARS-CoV-2 spike protein sensing using surface functionalized glassy carbon electrode

## Introduction

The highly infectious coronavirus disease (COVID-19) caused by severe acute respiratory syndrome coronavirus 2 (SARS-CoV-2) has seriously threatened human health and economic well-being worldwide for the last 3 years. There are four major structural proteins found within the single-stranded RNA SARS-CoV-2 virus: membrane (M), envelope (E), spike (S), and nucleocapsid (N).^1,2^ The virus’s membrane-attached spike proteins bind to the transmembrane angiotensin-converting enzyme-2 (ACE2) receptor and initiate cell entry to infect the host. Furthermore, recent studies have shown that the S protein plays a vital role in fusion, entry, and antibody production within host cells.^3^

A significant effort was launched worldwide to develop a “cure” for COVID-19 infections. However, therapy for COVID-19 infections is still undergoing clinical trials. In these circumstances, the world population depends on vaccines and quarantine to prevent the spread of SARS-CoV-2. Detecting coronavirus infections using a rapid, sensitive, and cost-effective technique to eliminate biosecurity threats has become essential. The growth of rapid tests, consistency, and hassle-free applicability at low cost is needed for the COVID-19 pandemic. Several research groups have developed numerous effective tests, including a molecular test for detecting SARS-CoV-2.^4–6^ Molecular tests are carried out using nose or/and throat swabs to detect viral genes. The samples are tested by real-time reverse transcription polymerase chain reaction (RT‒PCR).^7–10^ RT‒PCR, which is performed by skilled technical personnel in a laboratory-based medical establishment, is the most successful and most popular method for detecting SARS-CoV-2 infection.^5–11^ However, RT‒PCR also suffers from a high false-negative rate of 20% to 67% due to sampling error and the poor sensitivity of detection kits.^12,13^ Phuong Q. M. Nguyen et al. fabricated a modular microfluidic-based RT–PCR and RT-LAMP platform, Epidax^®^29, to detect SARS-CoV-2. They proposed an easily rearrangeable platform to perform COVID-19 screening by either endpoint RT–PCR or RT-LAMP tests or by confirmatory tests by real-time RT–quantitative PCR (RT–qPCR). A performance evaluation of its ability to detect SARS-CoV-2 was conducted using their fabricated endpoint RT‒PCR on 81 and 43 clinical RNA extracts. They established the rapid direct RT–PCR detection of SARS CoV-2 in 42 nasopharyngeal swab samples without RNA extraction; as a result, the sample-to-result testing time was shortened to 1 h.^4^

Many other diagnostic methods have also been proposed for SARS-CoV-2 detection, including those based on a lateral flow assay (LFA), lateral flow immunoassay (LFIA), enzyme-linked immunosorbent assay (ELISA), plasmonic sensors computed tomography (CT) imaging and electrochemical biosensing technologies.^2,6,14–23^ The most popular and commercially used method is ELISA-based detection, although it has certain drawbacks in detecting SARS-CoV-2. SARS-CoV-2 detection at low levels is tedious due to the complexity of the assay procedure and inadequate sensitivity. Most of the available kits for COVID-19 have high limits of detection (LODs) and result in high false-negative outcomes.^24^ ELISAs, LFAs and LFIAs provide significant advantages, being cost-effective, easy and relatively fast to execute, but suffer due to low sensitivity, resulting in a large number of false-negative results. Plasmonic sensor-based processes are sensitive and low in cost, but these instruments are unsuitable for field testing because they require specific biological labels and wide-ranging biochemical processing.^21,22^ CT imaging is inappropriate for early findings and is not easy to use for onsite testing.^22,25^

Consequently, general electrochemical approaches are sensitive, rapid, and economical with faster analysis capability and ease of use. They involve less complicated measurements than their optical counterparts and can work in complex analytes.^26–28^ In addition to being independent of optical interference, they require a minimum amount of sample, consume little power and are extremely well-matched with modern microfabrication techniques.^29–31^ Electrochemical biosensors can be transformed into point-of-care testing devices using low-cost electrode-based sensors that can be integrated with an easy, inexpensive, and portable handheld device for rapid detection.^32^

Andrea Idili et al. fabricated an electrochemical aptamer-based sensor for the detection of the SARS-CoV-2 spike protein. In their study, fluorescence spectroscopy was used to validate the ability of the designated aptamer to undergo conformational changes in the presence of its target. Using the DNA aptamer, the suggested fabricated sensor can identify picomolar levels of the SARS-CoV-2 spike protein in buffer, serum, and 50% artificial saliva. In the reported work, the authors proposed a single-step process that permits the target to be found in 15 s due to the fast binding kinetics of the aptamer.^33^

In recent work, Muhammad Adeel et al. developed a sensor using a precisely limited exfoliation of a flexible graphitic carbon foil for an antibody immobilization matrix. The fabricated electrodes were functionalized with ethylenediamine by covalent attachment, utilizing 1-ethyl-3-(3-dimethlaminopropyl) carbodiimide and N-hydroxysuccinimide activators. They suggested a new low-cost electrochemical sensor for detecting SARS-CoV-2 spike protein within 30 min with an extensive concentration range of 0.2–100 ng/mL. Detection limits of 25 pg/mL have been found in the case of phosphate buffer solution (pH 7.4) and 27 pg/mL for diluted blood plasma.^34^

Several processes are reported that require a significantly longer assessment span, but the technique established by Biljana Mojsoska et al. using a monoclonal anti-spike antibody infused into SPE provides rapid detection of SARS-CoV-2 spike protein.^35–38^ Nucleocapsid proteins were used in their electrochemical sensor, which took 25 h to prepare and detected 0.8 pg/mL nucleocapsid protein in 30 min.^39^ Rebeca M. Torrente-Rodrıguez et al. proposed a method involving immunosensor studies using SARS-CoV-2 spike and nucleocapsid antibodies and is capable of detection in less than 2 h.^40^ Laura Fabiani et al. determined SARS-CoV-2 spike or nucleocapsid antibodies in 30 min, but the LODs were high, in the ng/mL range.^41^ This shows that the development strategies for these biosensors are complicated and laborious. The best available system in the market to date is the Cobas Liat system of Roche Molecular Systems, Inc., which is capable of detecting SARS-CoV-2 in 20 min with high accuracy using RT‒qPCR.^42^ The LOD of Cobas Liat is ~125 copies of viral RNA/mL.

Although electrochemical biosensors have attracted significant attention for the detection of the SARS-CoV-2 spike protein due to their sensitivity and miniaturization capabilities, interference and selectivity problems cannot be ignored. Most of the abovementioned references did not specifically mention the interference and selectivity problems. We have addressed interference and selectivity in our work and reported the results in detail. These problems can be avoided if the following factors are considered:

1. Pretreatment steps, such as filtration or dilution of biological samples, including saliva, serum, or nasal swabs, may be necessary to minimize interference with SARS-CoV-2 detection.
2. Strategies such as surface functionalization, blocking agents, or surface modifiers may be employed to reduce nonspecific binding.
3. Careful selection of specific recognition elements, such as antibodies or aptamers, is important to minimize cross-reactivity and improve selectivity.
4. Signal amplification strategies such as enzyme-linked amplification or nanoparticle labels may enhance the selectivity and sensitivity of biosensors. This may also increase the detection limits and reduce the impact of interfering molecules.

Due to several limitations of the existing technologies, we attempted to develop a glassy carbon-based, inexpensive technique for detecting SARS-CoV-2 infection using an antibody-antigen binding technique. Glassy carbon (GC) has excellent electrochemical stability, low background noise, and advantageous biocompatibility.^43–45^ GC derived from a pyrolyzed photoresist film is an electrically conductive material (resistivity 5 × 10^−4^ Ωcm) and has a low surface oxygen level, possibly due to noncarbon atom termination from the polymer chain during pyrolysis.^46,47^ The current work describes a simple, advantageous immobilization procedure for rapidly detecting SARS-CoV-2 infection based on covalently linking the SARS-CoV-2 antibody to the GC surface without requiring a secondary antibody or label. Glutaraldehyde was used as a cross-linker between covalent attachments of glutaraldehyde with GC and was investigated using Fourier transform infrared spectroscopy (FTIR) characterization and cyclic voltammetric coefficient of variation (CV) analysis. To determine the effectiveness and sensitivity of the sensor electrode, electrochemical impedance spectroscopy (EIS) was utilized to measure the change in total impedance before and after incubation of the SARS-CoV-2 antibody with various concentrations of SARS-CoV-2 spike protein.

The Materials and Methods are presented in Section II of this paper, which includes the fabrication and characterization of electrodes, preparation of solutions, electrode surface modification, electrochemical measurements and FTIR characterization. The Results and Discussion are presented in Section III, including detailed explanations of the results obtained. A schematic representation of the steps involved in the fabrication of the glassy carbon electrode (GCE) and functionalization process for the SARS-CoV-2 spike protein electrochemical sensing platform is shown in Fig. 2.

## Materials and methods

### Reagents and solutions

Recombinant human coronavirus SARS-CoV-2 spike glycoprotein S1 (active) (ab273068) was utilized as the target molecule and was purchased from Abcam (Waltham, USA) (1 mg/ml). Recombinant Anti-SARS-CoV-2 Spike Glycoprotein S1 Antibody [CR3022] - Chimeric (ab273074) (Rabbit monoclonal) was also purchased from Abcam (Waltham, USA). For performing the experiments, a stock solution of SARS-CoV-2 in PBS (pH 7.4 and 100 mM ionic strength) was prepared and diluted to various concentrations from 1 fg/ml to 1 µg/ml. To evaluate the selectivity of the sensor toward the SARS-CoV-2 spike protein, hepatitis-B surface antigen (HBsAg) was tested at a concentration of 1 fg/ml. Recombinant Hepatitis-B virus and Hepatitis-B Surface Antigen AD protein (ab193473) procured from Abcam (Waltham, USA) (1 mg/ml) were used for the selectivity test. For the interference experiments, a solution of Hepatitis-B Surface Antigen in PBS (pH 7.4 and 100 mM ionic strength) was prepared and diluted to various concentrations from 1 fg/ml to 10 ng/ml. A prostate-specific antigen (PSA), a target molecule, was also purchased from Sigma Aldrich, USA (1 mg/ml) and used for interference experiments. The PSA solution at pH 7.4 and 100 mM ionic strength was serially diluted in PBS to obtain concentrations ranging from 1 pg/ml to 3 µg/ml. Dulbecco′s modified Eagle′s medium (DMEM) with high glucose medium was also used for selectivity testing.

### Fabrication of electrodes

**Fig. 1:**
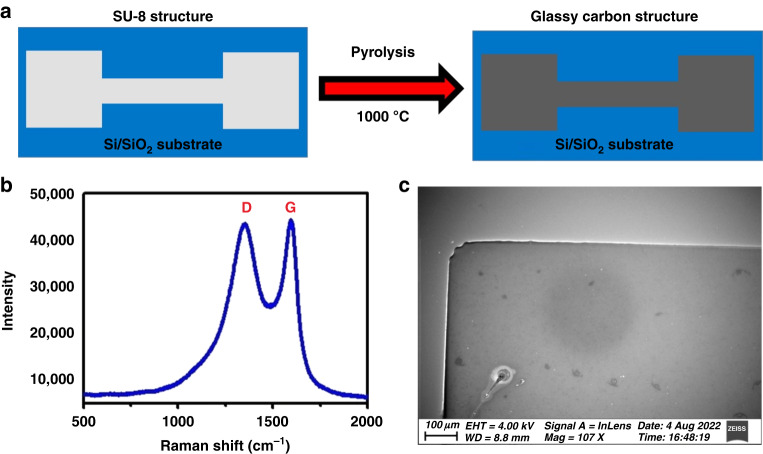
Schematic of the glassy carbon electrode fabrication and its characterizations. **a** Graphic representation of the transformation of SU-8 electrode to GCE. **b** Raman spectroscopy result of unmodified GCE. **c** SEM image of the GCE surface modification followed by electrochemical sensing

The conventional photolithography process was used to prepare SU-8 electrodes, followed by a pyrolysis process to convert the polymer structures into GC. We present a schematic of the conversion of the SU-8 electrode to a carbon electrode in Fig. 1a. Here, two electrode pads of 3 mm × 3 mm area are connected through a 5 mm-long and 1 mm-wide strip. One pad is assigned for electrical connection and the other for electrochemical sensing in the bare condition or in the functionalized state. We followed the same fabrication steps already published by our group.^48^

### Characterization of electrodes

The fabricated GCE was characterized using several techniques, including scanning electron microscopy and Raman spectroscopy. Extensive work on the impact of pyrolysis process parameter variation has been reported elsewhere.^48,49^ A schematic illustration of the conversion of SU-8 electrodes to carbon electrodes is shown in Fig. 1a. It is designed so that a stripline connects two electrode pads. One of the two pads makes the electrical connection, whereas the other is used for electrochemical sensing.

### Electrochemical measurement

An electrochemical analyzer from CH Instruments, CHI-760E, was used to characterize the bare GCE and biomodified electrodes. All electrochemical experiments were carried out under “no flow” conditions, and the measurements were carried out using 10 mM potassium ferrocyanide, K_4_[Fe(CN)_6_], in 0.5 M potassium chloride (KCl). CV measurements were performed using a similar setup to the three-electrode system at a scan rate of 10 mV/sec in a potential range of –0.6 V to 0.6 V using an Ag/AgCl electrode as a reference. For the EIS measurements, a 3-electrode configuration was used with an applied sinusoidal signal of 5 mV amplitude in the frequency range of 1 Hz to 1 MHz. The GCE was used as the working electrode, platinum wire as the counter electrode, and Ag/AgCl electrode as the reference electrode. Nyquist impedance plots were recorded after the electrodes were exposed to phosphate buffer solution (PBS).

### Bio functionalization of electrodes

**Fig. 2:**
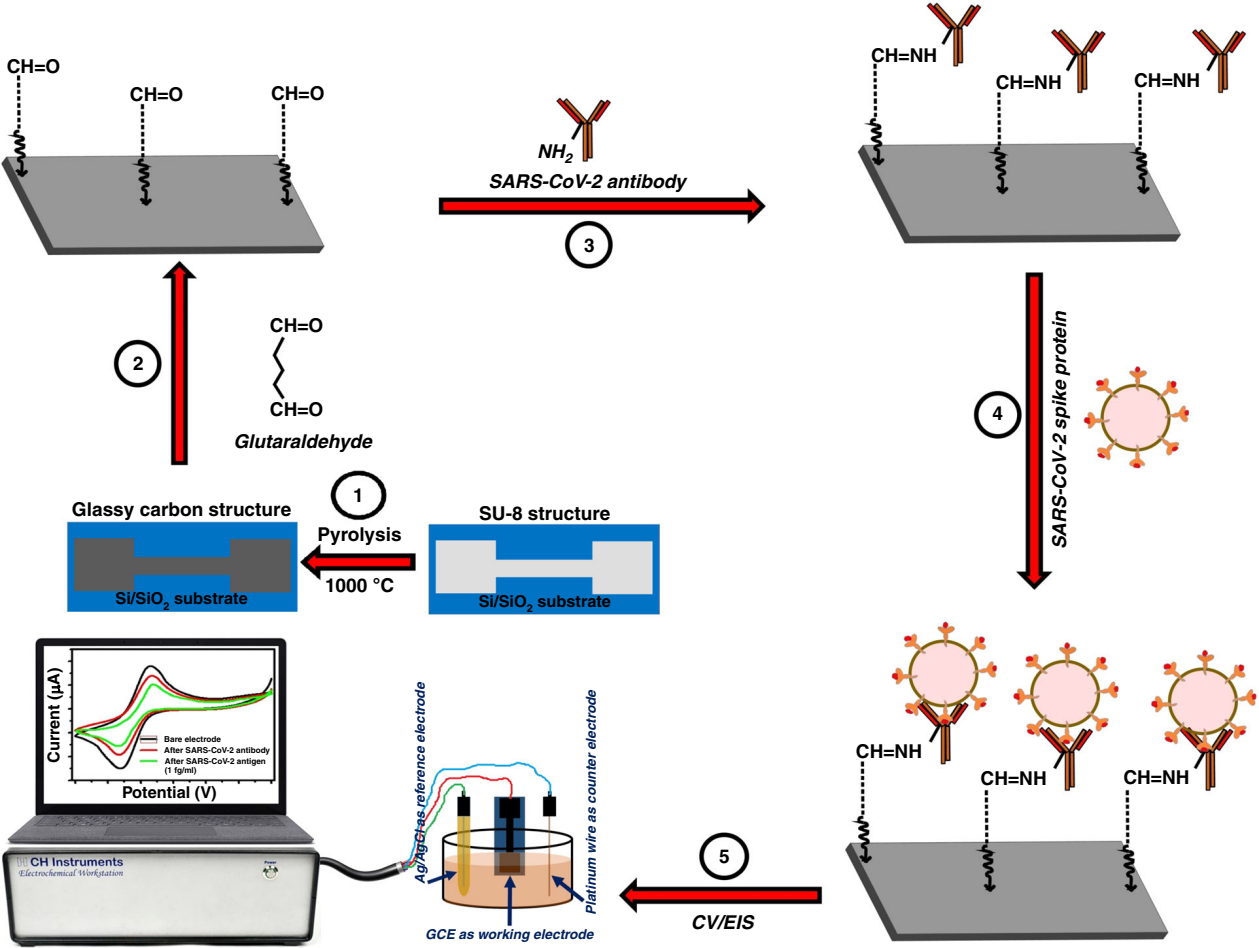
A schematic representation of the steps involved in fabrication of GCE and functionalization process for the SARS-CoV-2 spike protein sensing. Step 1: The photolithography process was used to prepare SU-8 electrodes, followed by pyrolysis process to convert the polymer structures into glassy carbon. Step 2: The GCE was treated with 4% glutaraldehyde aqueous solutions for 2 h. Step 3: The glutaraldehyde-modified GCE was incubated with the anti-SARS-CoV-2 antibody for 1 h. Step 4: GCE was incubated with different concentrations of SARS-CoV-2 spike protein solution (100 µL) for 20 min. Step 5: Electrochemical (CV/EIS) measurements were carried out

A schematic representation of the steps involved in the fabrication of the GCE and functionalization process for SARS-CoV-2 spike protein sensing is shown in Fig. 2. The fabricated GCE was treated with 4% glutaraldehyde aqueous solutions for 2 h and rinsed with deionized water to remove loosely bound glutaraldehyde from the surface. EIS measurements were carried out in each step to ensure the binding of glutaraldehyde with GC. To immobilize the anti-SARS-CoV-2 antibody, a glutaraldehyde-modified electrode was introduced to the anti-SARS-CoV-2 antibody. The reaction between the HC=O of glutaraldehyde and NH_2_ groups of antibodies via imine bond formation leads to immobilization of anti-SARS-CoV-2 antibody on the electrode surface. The glutaraldehyde-modified GCE was incubated with anti-SARS-CoV-2 antibody for 1 h and washed with phosphate-buffered saline (PBS). After antibody immobilization, the GCE was incubated with different concentrations of spike protein solution (100 µL) for 20 min, and EIS measurements were recorded to determine the change in total impedance after SARS-CoV-2 spike protein adhesion. The sensors were then exposed serially to higher concentrations of SARS-CoV-2 spike protein. The impedance values for different concentrations of SARS-CoV-2 spike protein are reported as the mean of three trials of each concentration.

### FTIR characterization

FTIR studies were carried out to characterize the bare electrode and modified electrode samples using a SHIMADZU (IRAFFINITY 1 S WL model) IR spectrometer with a resolution of 4 cm^−1^ in the 400–4000 cm^−1^ region. This instrument was equipped with Specac attenuated total reflection fitted with diamond crystal, scanned (transmittance vs. wavenumbers mode) at room temperature. Fourteen hundred scans were taken for accuracy, and the average value was reported.

## Results and discussion

### Electrode characterization

Figure 1b shows the Raman spectroscopy result for pyrolyzed carbon structures. Approximately 60% shrinkage was observed along the structure’s thickness. Figure 1c shows the SEM image of the GCE. In this sample, we see two dominant bands: a disordered band (D-band), located at ~1392 cm^−1^, and an ordered band (G-band), located at ~1607 cm^−1^.^50^ Other results are reported elsewhere.^48^ The intensity ratio of the two dominant bands (I_D_/I_G_) is taken as a measure of the structural disorder. The lower the ratio relates to the higher crystallinity and the lower disordered nature of the carbon.^51^ Based on these results and our previous studies, the carbon microstructure is glassy in nature.^48,49^

### Stepwise characterization of the sensor electrode using CV

**Fig. 3:**
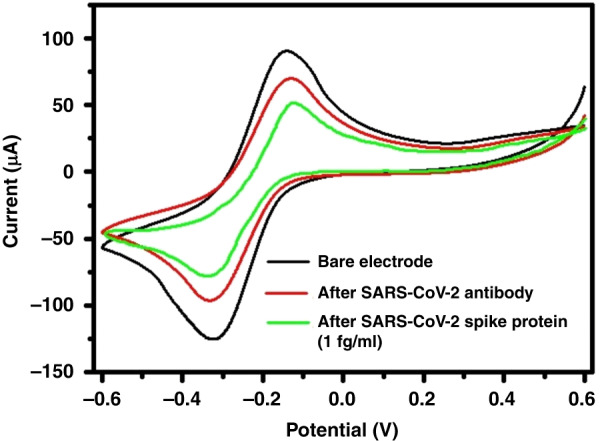
CV measurement for SARS-CoV-2 spike protein detection. Representative CV plots before and after SARS-CoV-2 spike protein immobilization with a concentration of 1 fg/ml; the measurement was carried out using 10 mM potassium ferrocyanide, K_4_[Fe(CN)_6_], in 0.5 M potassium chloride (KCl)

The CV plots for the bare electrode, electrode modified with SARS-CoV antibody, and electrode modified with SARS-CoV spike protein (concentration of 1 fg/ml) are presented in Fig. 3. After immobilizing SARS-CoV-2 antibodies and capturing the SARS-CoV-2 spike protein, we observed a decrease in current, which may be attributed to the reduction in the effective area of the working electrode. The electrodes have been observed to be capable of sensing down to 1 fg/ml. They can sense up to a concentration of 1 µg/ml. We calculated the LOD for the sensor to be ~31 copies viral RNA/mL.

### Characterization of the sensor electrode by FTIR

**Fig. 4:**
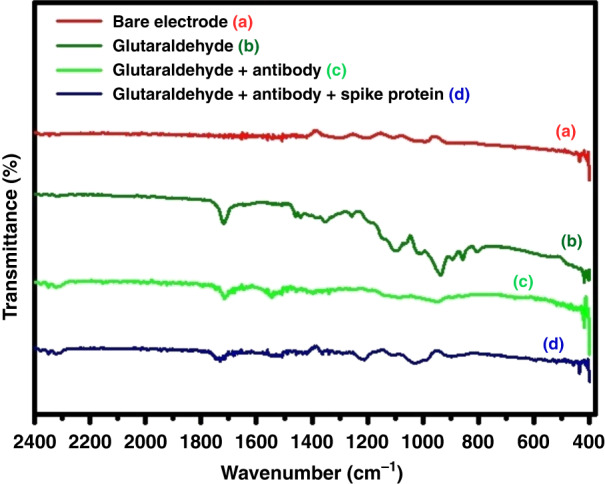
Surface modified glassy carbon electrode characterization using FTIR analysis. FTIR spectra of (**a**) Bare Electrode (**b**) Glutaraldehyde (**c**) Glutaraldehyde/antibody (**d**) Glutaraldehyde/antibody/spike protein electrodes

The GCE was treated with 4% glutaraldehyde aqueous solutions for 2 h. One end of the glutaraldehyde formed acetal or hemiacetal bonds with the surface hydroxyl groups (-OH) to become immobilized on the surface of the GC.^52,53^ After treatment, each sample was rinsed with deionized water to remove loosely bound glutaraldehyde from the surface, and FTIR measurements were carried out to understand the binding of GC with glutaraldehyde. A glutaraldehyde-modified electrode was reacted with the anti-SARS-CoV-2 antibody to form an imine bond with the aldehyde group of glutaraldehyde to immobilize the anti-SARS-CoV-2 antibody. The glutaraldehyde-modified GCE was incubated with the anti-SARS-CoV-2 antibody for 1 h, followed by washing with phosphate-buffered saline (PBS). FTIR measurements were carried out. After antibody immobilization, the GCE was incubated with different concentrations of SARS-CoV-2 spike protein solution for 20 min, and again, FTIR measurements were recorded. For each of the electrochemical processes, FTIR plots of transmittance vs. wavenumbers were recorded, as depicted in Fig. 4.

The unmodified GCE showed no peak at ~1800–1600 cm^−1^, as it does not possess any carbonyl functional (C=O) groups (Fig. 4a). After surface modification of glutaraldehyde, a peak at ~1717 cm^−1^ signified the presence of an aldehyde group (Fig. 4b). FTIR analysis of bare GCE could not confirm the presence of surface -OH groups, possibly due to the low concentration of sparsely dispersed -OH groups over the GCE surface. Further surface modification with the anti-SARS-CoV-2 antibody only decreased the intensity of the 1717 cm^−1^ peak, indicating a decrease in the number of aldehyde groups due to imine bond formation with the anti-SARS-CoV-2 antibody (Fig. 4c). However, no prominent imine band was visible at ~1690–1640 cm^−1^. Once the spike protein was added to the antibody-modified electrode, the peak at ~1714 cm^−1^ showed multiple shoulders at ~1732 and 1745 cm^−1^. All FTIR spectra indicate that electrode modification was indeed achieved.

### Detection of SARS-CoV-2 spike protein by EIS

**Fig. 5:**
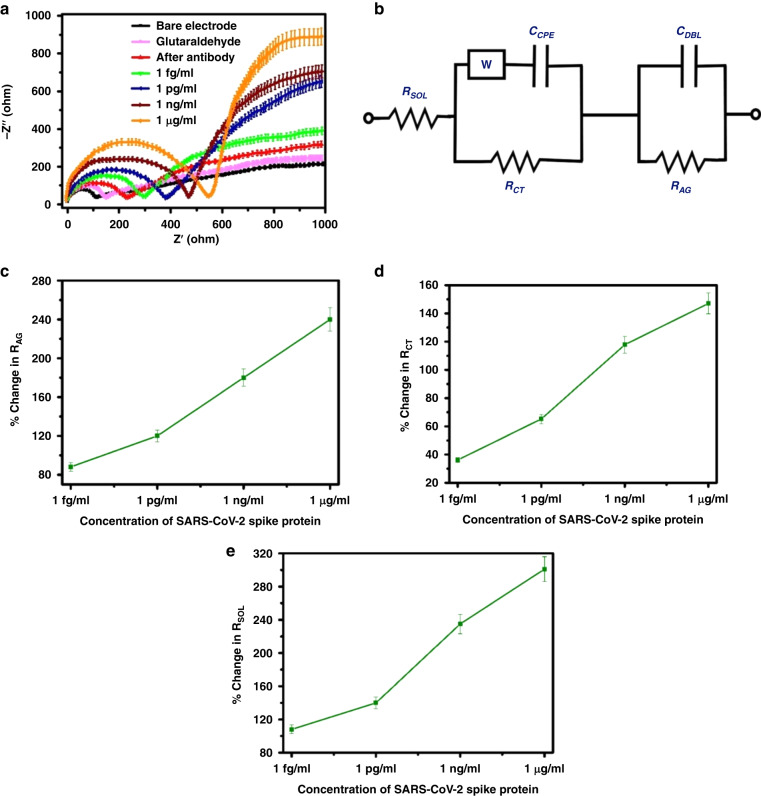
EIS measurement results. **a** Nyquist impedance plots of GC sensor electrode before and after capturing 1 fg/ml to 1 µg/ml of SARS-CoV-2 spike protein. **b** Equivalent circuit diagram. **c** R_AG_ Vs SARS-CoV-2 spike protein concentration. **d** R_CT_ Vs SARS-CoV-2 spike protein concentration. **e** R_SOL_ Vs SARS-CoV-2 spike protein concentration

We performed a series of EIS experiments to assess the effect of successive modifications of the electrode on its impedance spectrum. The AC impedance spectra are analyzed by generating a Nyquist plot (*Z*′, real impedance, versus *Z*′′, imaginary impedance) with CH Instruments software. EIS spectra of the bare and modified electrodes were obtained at the formal potential of the redox couple with an applied sinusoidal signal of 5 mV amplitude. We conducted our measurements over a frequency range of 1 Hz to 1 MHz, as shown in Fig. 5a, and fitted them with an equivalent circuit (Randles circuit), as shown in Fig. 5b. The diffusion coefficient of the SARS-CoV-2 spike protein can be calculated using the following Stokes-Einstein relationship:

1 $$D=\frac{{\rm{k}}{\rm{{\rm T}}}}{3{\rm{\pi }}{\rm{\eta }}{D}_{{\mathsf{H}}}}$$

where *k* is the Boltzmann constant, *Τ* is the absolute temperature (298 K), *η* is the medium viscosity, and *D*_*H*_ is the hydrodynamic diameter (15 nm). From Eq. (1), the calculated diffusion coefficient value at 298 K is 3.3 × 10^−7^ cm^2^ s^−1^; therefore, within 20 min, the sensor showed steady responses.^54^ Model parameters include bulk solution resistance (*R*_SOL_), double layer capacitance (*C*_DBL_) created by the ions at the electrode interface, charge transfer resistance (*R*_CT_), which represents the current flowing through this interface due to redox reactions, constant phase element (*C*_CPE_), and resistance of the attached SARS-CoV-2 spike protein molecules to the GC (*R*_AG_). In this case, the Warburg impedance (W) is an insignificant contribution to the model, and its value is relatively low. An ideal Nyquist plot displays a semicircle with a diameter followed by an approximately diagonal straight line. This semicircle represents the charge transfer resistance due to redox reactions at the interface with the electrode, and the straight line represents the impedance of the current due to diffusion from the solution to the interface. When specific or nonspecific analytes bind, the diameter of the semicircle increases, which decreases the surface area accessible for redox reactions. This may be due to SARS-CoV-2 spike protein caught at the site of the antibody obstructing the electron transfer process. The semicircle diameter correlates with the *R*_CT_ value, which was expected to increase at every successive step of electrode surface modification. As shown in Fig. 5c–e, the fitted values obtained from Nyquist graphs were presented. Notable changes were found in the *R*_CT_, *R*_AG_, and *R*_SOL_ values, which can be credited to the remarkable bridging of the gap between GCEs after SARS-CoV-2 spike protein capture.

### Impedance, sensitivity and selectivity measurement

**Fig. 6:**
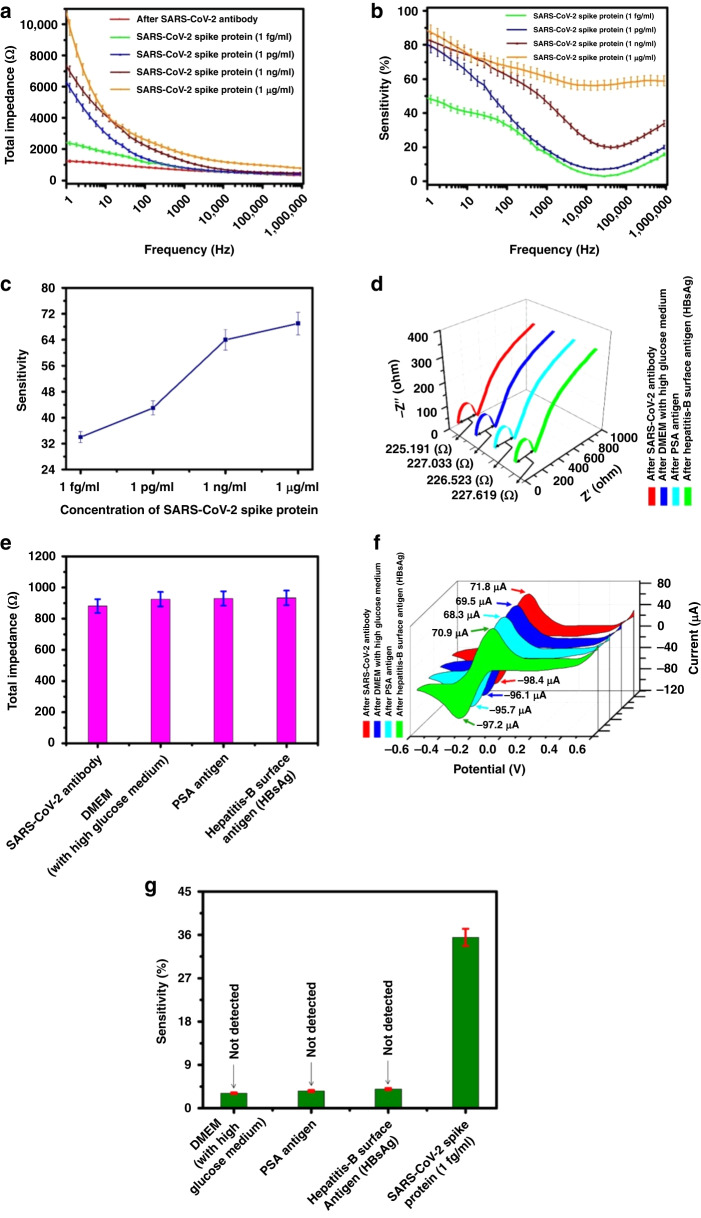
Sensitivity and selectivity performances of the developed sensor. **a** Total impedance variation with different frequency before and after exposure to four increasing concentration of SARS-CoV-2 spike protein (i) SARS-CoV-2 antibody, (ii) 1 fg/ml, (iii) 1 pg/ml, (iv) 1 ng/ml, (v) 1 µg/ml. **b** The change in average sensitivity characteristics with the frequency of the GCE-based sensor at an applied voltage of 5 mV for the sample containing concentrations of 1 fg/ml to 1 µg/ml SARS-CoV-2 spike protein. **c** The change in average sensitivity characteristics with varying SARS-CoV-2 spike protein concentrations at a frequency of 100 Hz. **d** Nyquist impedance plots of GC sensor electrode before and after capturing 1 fg/ml concentration of Hepatitis-B surface antigen, 3 µg/ml concentration of PSA antigen and DMEM with high glucose medium. **e** Plot column chart of total impedance at a specific frequency of 100 Hz in DMEM (with high glucose medium), concentration of PSA antigen (3 µg/ml), and Hepatitis-B surface antigen (1 fg/ml). **f** CV plots before and after capturing 1 fg/ml concentration of Hepatitis-B surface antigen, 3 µg/ml concentration of PSA antigen and DMEM with high glucose medium. The measurement was carried out using 10 mM potassium ferrocyanide, K_4_[Fe(CN)_6_], in 0.5 M potassium chloride (KCl). **g** Average sensitivity plots of the proposed sensor at a specific frequency of 100 Hz for 1 fg/ml concentration of Hepatitis-B surface antigen (HBsAg), 3 µg/ml concentration of PSA antigen, DMEM with high glucose medium and 1 fg/ml concentration of SARS-CoV-2 spike protein

The sensor sensitivity was evaluated by varying the concentration of the SARS-CoV-2 spike protein. The total change in the sensor’s impedance was calculated using the frequency range 1 Hz to 1 MHz and an AC amplitude of 5 mV. The change in total impedance [*Z* = (*Z*^2^_real_ + *Z*^2^_ima_)^1/2^] with frequency after SARS-CoV-2 antibody immobilization and different concentrations of SARS-CoV-2 spike protein capture is shown in Fig. 6a. In this work, multiple sensor electrodes were used to measure multiple concentrations of spike protein samples to prevent any interference from previous measurements. Three measurements were recorded to determine run-to-run variations and to compute sensitivities. Figure 6b shows the average sensitivity of each spike protein concentration for these three sensitivities. After exposure to four increasing concentrations of SARS-CoV-2 spike protein, the total impedance change with different frequencies was measured (presented in Fig. 6b). The sensitivity, S, was calculated as the fractional change in impedance expressed as a percentage, given in Eq. (2):

2 $${\rm{S}}=\frac{{Z}_{N}-{Z}_{{\rm{B}}}}{{Z}_{{\rm{B}}}}\,{\rm X}100$$

where ${Z}_{{\rm{N}}}$ signifies the total impedance value after attachment of the SARS-CoV-2 spike protein, and ${Z}_{{\rm{B}}}$ signifies the total impedance value after attachment of the SARS-CoV-2 antibody.

Figure 6b shows the change in the average sensitivity characteristics with the frequency of the GCE-based sensor at an applied voltage of 5 mV for samples containing concentrations of 1 fg/ml to 1 µg/ml SARS-CoV-2 spike protein. Figure 6c shows how the average sensitivity changes with varying SARS-CoV-2 spike protein concentrations at a frequency of 100 Hz.

A significant increase in the total impedance value at low frequency after SARS-CoV-2 spike protein capture, which indicates an increase in the charge transfer resistance (*R*_CT_), controls the total impedance value in this low-frequency range. A double layer also exhibits constant phase element behavior, which results in a drop in total impedance with frequency. When the SARS-CoV-2 spike protein concentration rises at lower frequencies, the percentage change in impedance is significant; however, when the SARS-CoV-2 spike protein concentration rises at higher frequencies, this percentage change becomes nonsignificant. When the frequency increases, the change in capacitive impedance becomes noticeable, thereby decreasing the overall increase in total impedance. Triplicate measurements were performed for each concentration and averaged to determine their sensitivities. For each concentration, the average sensitivity is shown in Fig. 6b. It has not been possible to reuse the sensors because there is some possibility that molecular interferences from previous antigens may be present on the sensor surface due to incomplete removal.

Figure 6b shows that for a concentration of 1 fg/ml, the sensitivity changes from 48% to 37% at a frequency of ~100 Hz. The amount gradually decreases with an increase in frequency. The decrease in the mid-frequency range (above 1 kHz) is ~10% for all concentrations of SARS-CoV-2 spike protein. SARS-CoV-2 spike protein concentrations below 1 fg/ml exhibit no statistically noticeable difference in impedance for any frequency range. A very small amount of SARS-CoV-2 spike protein molecules are captured below 1 fg/ml; the electron transfer process is not significantly impacted. At concentrations of more than 1 µg/ml, the electrode response tends to reach saturation, and it seems that once that point is reached, all the antibodies are occupied by the SARS-CoV-2 spike protein. The sensing ability of the electrodes has been demonstrated down to 1 fg/ml. We also calculated the *C*V number from EIS data at 100 Hz, which was found to be 0.398%.

Absorbance measurements were conducted to assess the intensity of antibody binding on the sensor electrode surface. Various concentrations of horseradish peroxidase (HRP) conjugated to human immunoglobulin G (IgG) (HRP-HIgG) antibodies were carefully dispensed onto the surface of the electrode. The subsequent absorbance readings were recorded, and these findings were previously documented in our earlier research.^49^ The sensor electrode underwent three rounds of rinsing with PBS to minimize any nonspecifically adsorbed antibodies, effectively removing any particles not specifically captured. Despite antibody immobilization, residual unreacted aldehyde groups might remain, which were blocked using BSA (bovine serum albumin) to prevent nonspecific antigen binding. Our previous studies showed that the highest antibody absorbance was achieved when utilizing an input concentration of 5 μg. In the investigation of antigen binding, a series of experiments were conducted involving the controlled application of alkaline phosphate-conjugated protein A using a range of concentrations. When antigen molecules were introduced onto the surface where antibody molecules were present, adsorption and desorption ensued until an equilibrium condition was achieved. In the beginning, the adsorption rate is higher, and over time, the desorption rate begins to rise.

The Scatchard equation provides the highest achievable antigen capture concentration.^55^

3 $$\frac{r}{c}={K}_{a}n-{K}_{a}r$$

In Eq. (3), $r$ represents the ratio of the concentration of bound antigen to the total concentration of antibodies, $c$ signifies the concentration of free antigens present, and $n$ denotes the number of binding sites available on each antibody molecule. The association constant ${K}_{a}$ quantifies the ratio of the forward association rate constant (${K}_{1}$) to the backward association rate constant (${K}_{-1}$), i.e., ${K}_{1}/{K}_{-1}$ = ${K}_{a}$. As the value of $n$ increases, the potential for binding more antigens also increases. Therefore, a higher ${n}$ value translates to an increased capacity for antigen binding, which can lead to a more efficient capture of antigens by the antibodies. In a specific antibody-antigen combination, the quantity of binding sites on individual antibody molecules, $n$, relies on the arrangement of molecules. The value of $n$ is influenced by how the molecules are positioned, and this positioning is affected by the initial treatment of the surface.^56^

The interaction between antibodies and antigens represents a particular chemical reaction facilitated through a process termed agglutination. The binding specificity is a result of the antibody’s paratope, located within the variable region of the polypeptide chain. This paratope specifically identifies antigenic determinants or epitopes. Remarkably, the variable regions within each antibody exhibit distinct amino acid sequences. These sequences play a pivotal role in enabling the precise binding of specific antigens. This binding process involves a complex interplay of various forces, including electrostatic interactions, hydrogen bonding, van der Waals forces, and hydrophobic interactions.^57^ This comprehensive array of mechanisms contributes to the precision and effectiveness of antigen recognition by antibodies. Additionally, the change in the absorbance with different antigen concentrations is described in detail in our research article published elsewhere.^49^ We used this information to estimate the quantity of antibodies bound to the electrode surface and conducted all the experiments. To evaluate the selectivity of the fabricated GCE sensor, it was exposed to 1 fg/ml hepatitis B surface antigen, 3 µg/ml PSA antigen and DMEM with high glucose medium. Figures 6d, f represent the Nyquist diagrams and the CV plots, respectively. It has been observed that the magnitude of the current change is within 4%, which is less than that of the specific antigen. Figure 6e represents the total impedance values at a specific frequency of 100 Hz of DMEM (with high glucose medium), concentration of PSA antigen (3 µg/ml), and Hepatitis-B surface antigen (1 fg/ml). The selectivity of the GCE was assessed with a 1 fg/ml concentration of hepatitis-B surface antigen (HBsAg), a 3 µg/ml concentration of PSA antigen and DMEM with high glucose, as shown in Fig. 6g. The average sensitivity shown in Fig. 6g was calculated at a specific frequency of 100 Hz using the formula provided in Eq. (2), ${Z}_{B}$ taking the total impedance value subsequent to the attachment of SARS-CoV-2 antibodies. It was tested against various potential interfering agents, such as DMEM with a high glucose medium, PSA antigen, and Hepatitis-B surface antigen, to evaluate the specificity of the biomodified GCE sensor. All of these components were introduced separately to the surface of the different GCEs. The GCE had previously been coated with anti-SARS-CoV-2 antibodies. In this context, ${Z}_{{\rm{N}}}$ represents the total impedance readings corresponding to particular interfering substances at a specific frequency of 100 Hz. The sensitivity was within 6% at a 1 fg/ml concentration of hepatitis B surface antigen, within 6% at a 3 µg/ml concentration of PSA antigen, and within 5% in DMEM with high glucose medium, which is very low compared to the 1 fg/ml concentration of SARS-CoV-2 spike protein. As observed, the responses to DMEM with high glucose medium, PSA antigen, and Hepatitis-B surface antigen show a change in total impedance value (~6% increase) compared to the total impedance of anti-SARS-CoV-2 spike glycoprotein S1 antibodies. This indicates a degree of interaction between the sensing structure and the selected interfering proteins, implying that their presence in the sample could impact the accuracy of virus quantification. Nevertheless, this value is notably less than the total impedance exhibited by the SARS-CoV-2 spike protein (1 fg/ml). This is primarily due to the strong binding capability of the biorecognition antibody components, specifically the anti-SARS-CoV-2 spike glycoprotein S1 antibodies, and the resistance of the sensing surface to interactions with the selected interfering substances.

We compared the performance of the developed sensor with recent reports on carbon-based SARS-CoV-2 spike protein biosensors and present them in Table 1. Our reported biosensors show a detection limit at least one order of magnitude lower than that of the most sensitive electrochemical biosensors. In addition, the sensor fabrication is highly scalable.

**Table 1:** An evaluation of the proposed GCE sensor in comparison to previous studies

References	Target	Sensor material	Sensing mechanism	Preparation & assay reaction time (estimated)	Assay sample-to-result time (estimated)	Detection limit (LoD)	Approximate cost (US $)
^35^	SARS-CoV-2 spike protein	Graphene	Field-effect transistor (FET)	>6 h	48 h	1 fg/mL	Not reported
^53^	SARS-CoV-2 spike protein	Carbon, filter paper, phenolic paper circuit board material, Nafion	Electrochemical	>4 h	4 min	2.8 fg/ml	4.67
^21^	SARS-CoV-2 spike protein	gold nanoislands (AuNIs)	Plasmonic photothermal	>4 h	>5 h	0.22 pM	Not reported
^22^	SARS-CoV-2 spike protein	toroidal dipole resonant meta molecules	Plasmonic biosensor	>5 h	~80 min	4.2 fM	Not reported
^38^	SARS-CoV-2 spike protein	graphene	Electrochemical	18 h	45 min	5.5 × 10^5^ PFU/mL	Not reported
Present work	SARS-CoV-2 spike protein	C-MEMS derived Glassy Carbon	Electrochemical	~3 h	~22 min	1 fg/ml	2

## Conclusions

There are very few existing technologies suitable for detecting asymptomatic cases of SARS-CoV-2 detection, which is one of the primary reasons for the spread of the virus. Therefore, we have developed an inexpensive technique for detecting SARS-CoV-2 infection using an antibody-spike protein binding method. C-MEMS-derived GC is used as a biosensor, and we describe a simple, advantageous immobilization procedure for rapidly detecting (<20 min) SARS-CoV-2 infection. The backbone of this work is covalently linking the SARS-CoV-2 antibody to the GC surface by using glutaraldehyde as a cross-linker. We investigated the attachment using FTIR characterization and CV analysis and found proper binding. We also used EIS to measure the change in total impedance before and after incubation of the SARS-CoV-2 antibody with various concentrations of SARS-CoV-2 spike protein. The sensor can sense 1 fg/ml to 1 µg/ml SARS-CoV-2 spike protein. The LOD was calculated to be ~31 copies of viral RNA/mL. The *C*V was calculated from EIS data at 100 Hz and found to be 0.398%. A fitting model of the equivalent electrical circuit was also used to explain the electrical behavior of the GCE-based biosensor. The developed sensor is usable and economical for mass screening.

**Fig. 7:**
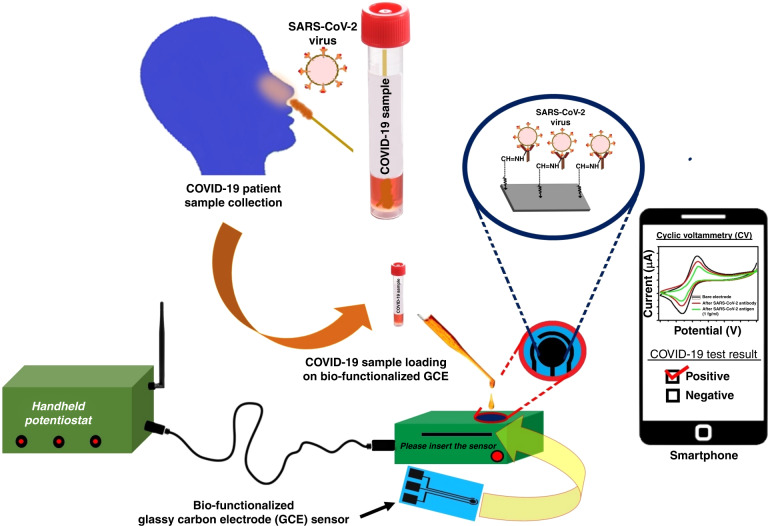
Schematic of the SARS-CoV-2 virus detection process using Point-of-Care device

We observed the capability of C-MEMS-derived GC to be used as a biosensor, as the surface modification technique makes it able to detect infection. We planned to use nasopharyngeal swab samples, as the virus is characterized by the spike protein, to detect SARS-CoV-2 with a large dynamic range. The process includes the collection of nasopharyngeal swab specimens from suspected COVID-19-infected persons and storing them in a universal transport medium.^35^ We will collect samples and add them to the biofunctionalized GCE sensor (described in Section II (E) in Materials and Methods) for detection. The complete system includes a semiautomated handheld potentiostat that can be used with little to no technical knowledge. A smartphone can be interfaced with the potentiostat for data display and immediate results. A schematic is shown in Fig. 7.

We have already developed a tailored hardware and sensing software inbuilt system with an adjustable voltage range of up to ±4 V and a maximum current measurement capability of up to ±5 mA.^32^ The hardware incorporates user-selectable switches that require the application of electric pulses, CV and EIS protocols, as needed. This handheld potentiostat uses a smartphone application (a.apk file) to display and transmit the data. Any smartphone with an Android 7.0 or higher operating system can be used for this application. The user should select the appropriate analysis option following the app activation process via Bluetooth settings. The received potentiostat data will then be processed by the smartphone app, which will display the SARS-CoV-2 test results, either positive or negative. We are currently working on a functional prototype along with a multiplexing approach to SARS-CoV-2 detection. This approach allows the detection and differentiation of various viral components or variants within a single sample. By designing specific primers and probes that target different regions of the SARS-CoV-2 genome, it is possible to detect and distinguish multiple genetic markers or variants of the virus, including the spike protein, nucleocapsid protein, or other viral components, in a single test run. This method also makes it possible to detect specific mutations or genetic variants associated with different strains or lineages of SARS-CoV-2. The advantages of multiplexing for SARS-CoV-2 testing include increased testing efficiency, reduced turnaround time, and higher cost-effectiveness. Detecting multiple targets simultaneously enables comprehensive testing and characterization of the virus in a single assay, which is particularly advantageous in scenarios where large-scale testing or the identification of specific variants is needed.
